# Emergency Department Boarding for Psychiatric Hospitalization in Older Adults: Placement Challenges and Associated Risks

**DOI:** 10.5811/westjem.61704

**Published:** 2026-05-13

**Authors:** Victoria P. Schulte, Cristina Guasch, Angela Landerholm, Danilo Rojas-Velasquez

**Affiliations:** * Harvard Medical School, Beth Israel Deaconess Medical Center, Department of Psychiatry, Boston, Massachusetts; † Boston College, Boston, Massachusetts; ‡ Beth Israel Deaconess Plymouth Hospital, Department of Psychiatry, Plymouth, Massachusetts

## Abstract

**Introduction:**

Older adults are increasingly presenting to emergency departments (ED) with psychiatric emergencies amid limited inpatient psychiatric capacity, resulting in prolonged ED boarding. Our primary objective was to quantify ED boarding duration for older adults awaiting psychiatric hospitalization in community EDs. We examined whether longer boarding was associated with functional decline and physical restraint.

**Methods:**

We conducted a retrospective cohort study of ED encounters among adults ≥ 65 years of age who received a behavioral health evaluation by emergency services program (ESP) clinicians (non-prescriptive behavioral health professionals) who determined psychiatric level of care in two community EDs. The study period was from January 2023–June 2024. The primary outcome was boarding duration. We measured boarding duration from initiation of a psychiatric bed search to ED departure or psychiatric clearance. Secondary outcomes were functional decline (new loss of physical function that impaired activities of daily living, identified from serial nursing documentation during the ED stay) and physical restraint episodes.

**Results:**

Of 334 behavioral health encounters (mean age 76 years ± 7.4 years), 180/334 (53.9%) boarded ≥ 24 hours. The median boarding duration for psychiatric hospitalization was 44 hours (interquartile range 24–70). Functional decline occurred in 42/334 (12.6%) and restraint episodes in 27/334 (8.1%), with both events occurring only among encounters boarding ≥ 24 hours. Patients with neurocognitive disorders (157/334, 47.0%) had higher rates of functional decline (difference 17.1%, 95% CI, 7.3–26.4; *P* < .001) and restraint episodes (difference 11.2%, 3.0–19.1; *P* < .001) compared to patients without neurocognitive disorders.

**Conclusion:**

In this community ED cohort, older adults awaiting psychiatric hospitalization frequently experienced prolonged boarding, associated with higher rates of functional decline and physical restraint. Limitations include the retrospective design and reliance on nursing documentation to identify functional decline, with wide confidence intervals due to small event counts, limiting causal inference.

## INTRODUCTION

The emergency department (ED) is often the first point of contact for patients experiencing a psychiatric emergency. Psychiatric presentations account for 4–10% of ED visits, are more time-intensive, and more likely to result in hospital admission than non-psychiatric visits.^1^, ^2^ While psychiatric emergency visits have been increasing, the number of inpatient psychiatric beds has been declining,^3^ resulting in a limited resource that has become high in demand. As a result, many patients who require inpatient psychiatric care experience extended periods of ED boarding, a practice associated with adverse events (medication errors, increased morbidity/mortality), longer length of stay, and ED crowding.^4^, ^5^

Older adults are particularly vulnerable in this context because of the complexity of geriatric psychiatry emergencies and the limited number of specialized geriatric psychiatry units.^6^–^8^ Limited bed capacity is compounded by shortages of psychiatry clinicians, particularly in community settings. More than half of U.S. counties have no practicing psychiatrist, and over 100 million people live in federally designated mental health professional shortage areas, with disproportionately limited access in rural regions.^9^ In a national survey of ED directors by the American College of Emergency Physicians, 62% reported lacking dedicated psychiatric services for boarding patients.^10^ With these shortages, boarding becomes common, and older adults are more susceptible to the adverse effects of boarding as they have high risk of falls, delirium, and medication side effects (orthostasis, extrapyramidal symptoms) in the ED setting.^11^–^12^

Existing work has described the frequency and duration of ED boarding among adults with psychiatric illness,^13^, ^14^ and identified predictors of prolonged length of stay and adverse events in behavioral health-related ED visits.^15^, ^16^ Although ED psychiatric boarding is recognized, less is known about its frequency and consequences among older adults. In addition, few studies have examined geriatric-relevant outcomes such as functional decline and restraint use, particularly in community EDs that have less access to specialized psychiatry services. To address this gap, we conducted a retrospective cohort study of older adults undergoing behavioral health evaluation in two Massachusetts community EDs within the same healthcare system. Our objectives were to 1) quantify ED boarding duration for older adults awaiting psychiatric hospitalization and 2) evaluate associations between boarding duration and functional decline and restraint episodes. We hypothesized that the majority of older adults would experience prolonged boarding (≥ 24 hours) and that longer boarding would be associated with functional decline and physical restraint.

## METHODS

### Study Design and Setting

We performed a retrospective cohort study of ED encounters for adults ≥ 65 years of age who received a behavioral health evaluation at two community hospitals between January 1, 2023–June 30, 2024. The hospitals belong to the same integrated health system, share a single electronic health record (EHR), and share many of the same emergency clinician staff.

Hospital A is a 60-bed community hospital with approximately 24,000 annual ED visits. Hospital B is a 190-bed community hospital with approximately 44,000 annual ED visits and an on-site geriatric psychiatry unit (24 licensed beds, 10–12 typically staffed during the study period). Elsewhere in the system, a separate facility maintained a 15-bed geriatric psychiatry unit. Both EDs had weekday daytime consultation-liaison (C-L) psychiatry coverage from 8 am-5 pm. Weekend coverage consisted of telepsychiatry by a psychiatric nurse practitioner (8 am–12 pm) for new consults only. Although Hospital B has a geriatric psychiatry unit, this unit serves all hospitals within the integrated system. The psychiatrists and social workers on the geriatric psychiatry unit do not evaluate ED patients and are not part of the ED psychiatry consult team at either site. Accordingly, the availability of ED psychiatry consultation was comparable across the two hospitals; any potential site-level differences may relate to inpatient bed availability and disposition logistics rather than differences in ED consult staffing.

**Population Health Research Capsule**

What do we already know about this issue?

*Emergency department (ED) psychiatric boarding is common and associated with adverse events*.

What was the research question?

*What is the duration of psychiatric boarding in older adults, and is boarding associated with decline or physical restraint?*

What was the major finding of the study?

*Median ED boarding time was 44 hours (IQR 24–70), with 53.9% of encounters boarding ≥ 24 hours*.

How does this improve population health?

*Identifying prolonged boarding as a driver of geriatric harm supports targeted ED interventions to improve care of older adults with psychiatric emergencies*.

The Figure illustrates the typical workflow for ED patients receiving a behavioral health evaluation. For psychiatric emergencies, the first point of contact was an emergency services program (ESP) clinician, who performed the initial behavioral health evaluation within 60 minutes of an emergency clinician’s consult request. In this system, ESP evaluation is the required initial behavioral health assessment requested by emergency physicians for patients with psychiatric symptoms after initial medical screening. The ESP clinician determines psychiatric level of care and initiates the psychiatric bed search when indicated. The C-L psychiatry consultation is separate and is requested at the ED team’s discretion and clinical judgment. Reasons for consulting can include diagnostic clarification (e.g., delirium vs primary psychiatric disorder), complex medication management, or decision-making capacity questions. The C-L psychiatry consultation is not required for ESP evaluation and was available for both boarding and non-boarding patients. The C-L psychiatrists at both sites are part of the same psychiatry department within the larger healthcare system and, thus, have similar geriatric assessment protocols.

Bed searches for ED boarders were coordinated via the Massachusetts Behavioral Health Access registry, which is updated daily. The ESP staff listed boarding patients, particularly those with public insurance, and collaborated with the state Medicaid program to address lapses in coverage when present. Bed searches encompassed both health-system facilities and external hospitals. The ESP clinicians reassessed boarders daily to confirm continued need for admission and to consider alternate disposition options.

The institutional review board (IRB) approved this study as exempt because data were retrospective, de-identified, and posed minimal risk. All data were abstracted from the shared EHR using uniform documentation standards. We designed and reported the chart review in line with recommended safeguards for medical record review in emergency medicine,^17^ including pre-specified case selection and variable/outcome definitions, use of standardized abstraction forms with abstractor training/pilot testing, performance monitoring via dual abstraction, identification of the medical record source, description of the sampling frame, pre-specified handling of missing/conflicting data, and documentation of IRB status. Because abstractors were study investigators, blinding to study objectives was not feasible; interobserver agreement was addressed through dual abstraction and consensus but not quantified with a kappa statistic given the small calibration sample and low event counts. The study adheres to Strengthening the Reporting of Observational Studies in Epidemiology guidelines.^18^

### Variable Definitions

We pre-specified all variables and outcome measures before data collection. We first described ED boarding duration and the prevalence of prolonged boarding, defined as a patient remaining in the ED for > 24 hours after the initial behavioral health evaluation determined the need for psychiatric hospitalization. Boarding time was measured from the time the initial behavioral health evaluation was signed (which in this system is required for the psychiatric bed search to begin) to the time the ED discharge order was placed or when the patient was deemed to be psychiatrically cleared and no longer requiring inpatient psychiatric treatment.[Table t1-wjem-27-572]

Secondary outcomes included physical restraint episodes and functional decline. A restraint episode was defined as any documented use of physical restraints during the ED encounter. We focused on physical restraints because sedating medication (“chemical restraint”) could not be reliably classified from the medical record as restraint vs treatment, and pro re nata sedation documentation was inconsistent.

Because standardized functional scales (eg, modified Rankin Scale, Barthel Index) are not routinely collected as part of ED or behavioral health evaluations at these sites, we could not calculate numeric change scores for baseline vs 24-hour function. Instead, functional decline was operationalized as a new loss of physical function, documented by nursing staff during the ED stay, that impaired the patient’s ability to perform one or more basic activities of daily living (ADL) or instrumental ADLs. This included new or increased need for hands-on assistance with ambulation, transfers, toileting, or feeding. Abstractors reviewed serial nursing notes and flowsheets across the entire ED stay and coded functional decline as present only when a change from the patient’s prior status (either during that same ED visit or, when explicitly documented, their usual function at home or in a facility) was clearly described. If nursing documentation did not explicitly describe a change, functional decline was coded as absent, meaning that the patient was considered as having no functional decline present.

Potential confounders/covariates included age, sex (categorized as male, female, or other), and diagnostic category (neurocognitive disorder, primary psychiatric disorder, or substance use disorder). Diagnosis was based on the initial behavioral health evaluation, which is required to list a primary diagnosis and reason for presentation; primary psychiatric disorders included conditions such as schizophrenia, bipolar disorder, major depressive disorder, and other non-neurocognitive diagnoses. We did not perform a formal sample size calculation; to maximize power, we included all encounters for patients ≥ 65 years of age who received a behavioral health evaluation in the ED during the study period.

### Statistical Analysis

Patient characteristics were summarized as mean (SD) or median (IQR) for continuous variables and n (%) for categorical variables. Group comparisons used **χ**^2^ (or the Fisher exact test when sparse) and *t*-test or Wilcoxon rank-sum, as appropriate. For binary outcomes (prolonged boarding, functional decline, restraint), we report absolute risk differences (percentage-point differences) with 95% confidence intervals. Because functional decline and restraint events were sparse and included zero cells in some comparisons, we used the Fisher exact test for *P* values and calculated CIs for risk differences using the Newcombe method (Wilson score intervals). Two-sided α = 0.05. Wide CIs reflect sparse outcomes and zero cells in some comparisons rather than collinearity among covariates.

## RESULTS

There were 334 ED behavioral health evaluations for patients ≥ 65 years of age (mean age 76.1). Overall, 180/334 (53.9%) boarded ≥ 24 hours. Among the 189 encounters ultimately discharged to psychiatric hospitalization, the median boarding duration was 44 hours (IQR 24–70). Boarding duration did not differ significantly by primary diagnostic category, and age was not associated with boarding time in bivariate analyses.

Among encounters boarding ≥ 24 hours, functional decline occurred in 42/180 (23.3%) compared with 0/154 (0%) among encounters boarding < 24 hours (difference 23.3%, 95% CI, 15.3–30.9; *P* < .001). Physical restraint episodes occurred in 27/180 (15.0%) compared with 0/154 (0%) episodes among encounters boarding < 24 hours (difference 15%, 8.1–20.9; *P* < .001). Encounters with neurocognitive disorders had higher rates of functional decline (34/157 [21.7%]) than encounters without neurocognitive disorders (8/177 [4.5%]; difference 17.1%, 95% CI, 7.3–26.4; *P* < .001). Encounters with neurocognitive disorders also had higher rates of restraint episodes (22/157 [14.0%] vs 5/177 [2.8%]; difference 11.2%, 95% CI, 3.0–19.1; *P* < .001).

## DISCUSSION

In this two-site community ED cohort, more than half of encounters awaiting psychiatric hospitalization remained in the ED for > 24 hours with a median boarding duration of 44 hours (IQR 24–70). This shows that geriatric psychiatry boarders in community EDs often spend multiple days in an environment not designed for ongoing inpatient-level care. Our boarding durations appear longer than those reported in prior ED studies (Table 2), which have typically found median or mean stays in the 16–27 hour range for psychiatric presentations, and substantially longer than the three hours of boarding reported for older adults admitted to general medical services.^4^,^15^,^19^

### Functional Decline and Restraint as Geriatric Safety Outcomes

The rates of functional decline are notable given that detection relied on routine nursing documentation rather than systematic screening and, therefore, likely underestimate the true burden. Patients with neurocognitive disorders were particularly vulnerable to these outcomes. This aligns with broader literature showing that older adults with major neurocognitive disorders are highly susceptible to delirium, falls, and iatrogenic complications when immobilized or exposed to unfamiliar, overstimulating environments.^20^, ^21^ In our setting, only a small minority of prolonged boarders received physical therapy evaluation, and most mobility or functional concerns were triggered reactively when nurses documented decline, rather than through proactive screening. These observations reinforce that geriatric psychiatric boarding should be conceptualized as a period of elevated risk for functional loss, not merely “waiting time” prior to definitive treatment.

Restraint episodes were concentrated among prolonged boarders and frequently occurred in the context of agitation, which was also the most documented barrier to psychiatric placement. This suggests a self-reinforcing cycle: severe agitation contributes to difficulty with psychiatric placement; longer boarding increases the likelihood of restraint; and restraint itself may worsen agitation or precipitate injury in frail older adults.^16^

Boarding duration did not differ significantly across primary diagnostic categories, whereas neurocognitive disorders were strongly associated with functional decline and restraint. This pattern suggests that system-level constraints (bed availability, acceptance criteria) and geriatric vulnerability may be more important determinants of adverse outcomes than specific psychiatric diagnoses.

### Implications for Emergency Department Systems

Our findings support viewing older adults awaiting psychiatric admission as a distinct high-risk boarding group that warrants targeted ED protocols. First, early risk stratification is feasible using information available at the time of behavioral health evaluation: presence of a neurocognitive disorder, severe agitation or behavioral disturbance, and anticipated difficulty with placement. Patients with these features could be flagged for enhanced delirium-prevention and mobility interventions from the outset, rather than waiting until functional decline is documented.

Second, for patients expected to remain in the ED beyond 24 hours, structured “boarding bundles” could include scheduled ambulation when safe, orientation cues, sleep promotion, liberalized family presence, and explicit triggers for physical therapy or geriatrics consultation when new ADL dependence is observed. Drawing on work in general medical populations showing that even modest reductions in ED boarding can reduce downstream delirium and agitation,^12^,^19^ such bundles could be adapted and tested specifically for geriatric psychiatry boarders.

Third, our results highlight the need for diagnosis-informed agitation pathways that balance safety, preservation of function, and acceptability to receiving psychiatric units. This may include training staff to use non-pharmacologic strategies whenever possible, using appropriate medications for agitation management, and closely monitoring for side effects. Finally, increasing timely access to psychiatric expertise, through expanded daytime coverage, standardized triggers for consultation, or telepsychiatry, may help community EDs manage complex geriatric psychiatry presentations while bed searches are underway. Prior work suggests that telepsychiatry can improve access and streamline disposition in settings with limited on-site specialists.^22^^, 23^ In parallel, reducing boarding duration will require system- and policy-level changes such as expanding inpatient psychiatry bed capacity and strengthening regional bed market coordination, to increase timely access to appropriate psychiatric placement for older adults.

## LIMITATIONS

This study has several limitations. The retrospective design limits causal inference and is subject to unmeasured confounding. Outcomes were identified from routine clinical documentation (without standardized functional assessments); thus, misclassification is possible, and functional decline may be underestimated. In addition, the small sample size and low event counts resulted in wide confidence intervals. We did not measure baseline frailty or detailed medication exposure over time, which may influence both boarding duration and geriatric safety outcomes.

Generalizability is limited because this cohort was drawn from two community EDs within a single integrated health system in MA. Local/state-specific workflows for behavioral health evaluation and psychiatric bed searching may differ from other regions. We defined psychiatric boarding from completion of the behavioral health evaluation that initiates the bed search to ED departure or psychiatric clearance. Because boarding definitions vary across studies (e.g., ED arrival-to-departure or bed request-to-departure), direct comparisons of absolute boarding times should be interpreted cautiously. Nonetheless, the geriatric vulnerabilities highlighted here—functional decline and restraint during prolonged ED stays, along with limited access to specialty psychiatry services—are clinically plausible in any setting where older adults await psychiatric placement. The ED-level mitigation strategies discussed may be adaptable for other hospitals experiencing psychiatric boarding.

## CONCLUSION

This study extends the literature on ED psychiatric boarding by demonstrating that, among older adults in community EDs, prolonged waits for psychiatric admission are common and are linked to functional decline and restraint. These are outcomes with direct consequences for independence, safety, and quality of life. By identifying neurocognitive disorders, agitation, and boarding duration as markers of heightened risk, our findings point to concrete ED-level targets for delirium-prevention, mobility, and agitation-management strategies that can be tested in future work.

## Figures and Tables

**Figure 1:**
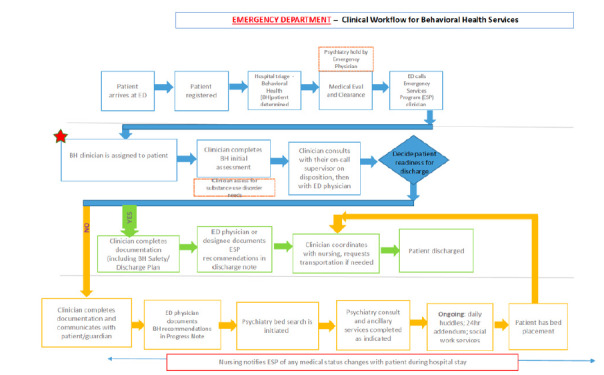
ED Behavioral Health Evaluation Workflow. *ED*, emergency department; *hr*, hour.

**Table 1 t1-wjem-27-572:** Sociodemographic and clinical characteristics of elderly patients awaiting psychiatric hospitalization in a study of the effects of prolonged boarding in the emergency department.

Variable	Mean (SD) or n (%)
Age, mean (SD), years	76.1 (7.4)
65–74	154 (46.1%)
75–84	114 (34.1%)
≥85	66 (19.8%)
Female, n (%)	164 (49.1%)
Race/Ethnicity, n (%)	
White non-Hispanic	289 (86.5%)
Black non-Hispanic	12 (3.6%)
Hispanic/Latino	12 (3.6%)
Asian	4 (1.2%)
Other/unknown	17 (5.1%)
Patient origin, n (%)	
Private residence	197 (59.0%)
Living alone	41 (12.3%)
Assisted living	37 (11.1%)
Skilled nursing facility	56 (16.8%)
Memory care unit	34 (10.2%)
Short-term rehab	7 (2.1%)
Group home	3 (1.0%)
Insurance, n (%)	
Medicare	212 (63.5%)
Medicaid	15 (4.5%)
Dual Medicare/Medicaid	84 (25.1%)
Commercial	23 (6.9%)
Mode of ED Arrival	
EMS	246 (73.7%)
Self-present	88 (26.3%)
Arrival Time	
Day (0700 – 1459)	136 (40.7%)
Evening (1500 – 2259)	148 (44.3%)
Night (2300 – 0659)	50 (15.0%)
Number of chronic medical conditions	
0–2	56 (16.8%)
3–4	142 (42.5%)
≥5	136 (40.7%)
Neurocognitive disorder	157 (47.0%)
MND, NOS	109 (32.6%)
Alzheimer dementia	19 (5.7%)
Vascular dementia	10 (3.0%)
Parkinson disease	14 (4.2%)
Lewy body dementia	5 (1.5%)
Primary psychiatric disorder	160 (47.9%)
Major depressive disorder	72 (21.6%)
Bipolar disorder	35 (10.5%)
Schizophrenia/schizoaffective	31 (9.3%)
Anxiety disorder	16 (4.8%)
Personality disorder	2 (0.6%)
Other	4 (1.2%)
Substance use disorder	17 (5.1%)
Patients on home psychiatric medications	174 (52.1%)
Antidepressants	127 (38.0%)
Antipsychotics	73 (21.9%)
Benzodiazepines	40 (12.0%)
Other	50 (15.0%)
Reason for behavioral health evaluation	
Agitation/behavioral disturbance	147 (44.0%)
Mood symptoms	62 (18.6%)
Psychosis	40 (12.0%)
Suicidal ideation or attempt	33 (9.9%)
Anxiety	17 (5.1%)
Other	35 (10.4%)

Disposition	n (%)

Psychiatric hospital	189 (56.6%)
Medical admission	27 (8.1%)
Private residence	68 (20.4%)
Assisted living	10 (3.0%)
Skilled nursing facility	20 (6.0%)
Memory care unit	14 (4.2%)
Short-term rehab	3 (1.0%)
Group home	3 (1.0%)

*ED*, emergency department; *EMS*, emergency medical services; *MND, NOS*, major neurocognitive disorder, not otherwise specified; *SD*, standard deviation.

**Table 2:** Boarding time compared to prior studies in a current study of the effects of prolonged boarding in the emergency department on elderly patients awaiting psychiatric hospitalization.

Study	Population	Boarding time, hours	Notes
Current study	Patients ≥ 65 years boarding for psychiatric admission	Median ED boarding time 44.6 hours (IQR 24–70)	2 community hospitals in MA with no academic psychiatry department
Simpson et al 2014	Patients ≥ 18 years boarding in psychiatric emergency room for psychiatric admission	Median ED boarding time 27.2 hours (0.3–143.0)	Psychiatric emergency room within an academic urban safety-net hospital
Rhodes et al 2016	Patients ≥ 65 years with primary psychiatric chief complaint	Median LOS 16.2 hours (9.7–29.7)	Community hospital Trauma Level III ED
Lai et al 2018	Patients age 60–89 years who received psychiatry consult in ED	Mean ED boarding time 27 hours (SD not given)	Academic, tertiary medical center
Joseph et al 2024	Patients ≥ 65 years who were admitted to general medical services	Mean ED boarding time 2.9 hours (SD 3.1)	2 academic hospitals and 5 community hospitals in MA

*ED*, emergency department; *IQR*, interquartile range; *LOS*, length of stay; *MA*, Massachusetts; *SD*, standard deviation.
